# Sensory and sympathetic nerve fibers undergo sprouting and neuroma formation in the painful arthritic joint of geriatric mice

**DOI:** 10.1186/ar3826

**Published:** 2012-05-01

**Authors:** Juan M Jimenez-Andrade, Patrick W Mantyh

**Affiliations:** 1 Department of Pharmacology, University of Arizona, 1501 N. Campbell Avenue, Tucson, AZ 85724, USA; 2 Unidad Académica Multidisciplinaria Reynosa Aztlán, Universidad Autónoma de Tamaulipas, Calle 16 y Lago de Reynosa, Reynosa, 88740, México; 3 Arizona Cancer Center, University of Arizona, 1501 N. Campbell Avenue, Tucson, AZ 85724, USA; 4 Research Service, VA Medical Center, One Veterans Drive, Minneapolis, MN 55417, USA

## Abstract

**Introduction:**

Although the prevalence of arthritis dramatically increases with age, the great majority of preclinical studies concerning the mechanisms that drive arthritic joint pain have been performed in young animals. One mechanism hypothesized to contribute to arthritic pain is ectopic nerve sprouting; however, neuroplasticity is generally thought to be greater in young versus old nerves. Here we explore whether sensory and sympathetic nerve fibers can undergo a significant ectopic nerve remodeling in the painful arthritic knee joint of geriatric mice.

**Methods:**

Vehicle (saline) or complete Freund's adjuvant (CFA) was injected into the knee joint of 27- to 29-month-old female mice. Pain behaviors, macrophage infiltration, neovascularization, and the sprouting of sensory and sympathetic nerve fibers were then assessed 28 days later, when significant knee-joint pain was present. Knee joints were processed for immunohistochemistry by using antibodies raised against CD68 (monocytes/macrophages), PECAM (endothelial cells), calcitonin gene-related peptide (CGRP; sensory nerve fibers), neurofilament 200 kDa (NF200; sensory nerve fibers), tyrosine hydroxylase (TH; sympathetic nerve fibers), and growth-associated protein 43 (GAP43; nerve fibers undergoing sprouting).

**Results:**

At 4 weeks after initial injection, CFA-injected mice displayed robust pain-related behaviors (which included flinching, guarding, impaired limb use, and reduced weight bearing), whereas animals injected with vehicle alone displayed no significant pain-related behaviors. Similarly, in the CFA-injected knee joint, but not in the vehicle-injected knee joint, a remarkable increase was noted in the number of CD68^+ ^macrophages, density of PECAM^+ ^blood vessels, and density and formation of neuroma-like structures by CGRP^+^, NF200^+^, and TH^+ ^nerve fibers in the synovium and periosteum.

**Conclusions:**

Sensory and sympathetic nerve fibers that innervate the aged knee joint clearly maintain the capacity for robust nerve sprouting and formation of neuroma-like structures after inflammation/injury. Understanding the factors that drive this neuroplasticity, whether this pathologic reorganization of nerve fibers contributes to chronic joint pain, and how the phenotype of sensory and sympathetic nerves changes with age may provide pharmacologic insight and targets for better controlling aging-related joint pain.

## Introduction

The prevalence of arthritis generally increases with age and is frequently accompanied by significant joint pain [1-5]. In individuals with arthritis (for example, rheumatoid arthritis (RA), osteoarthritis (OA)), joint pain generally decreases the functional status and quality of life, as it is strongly associated with physical disability, decreased mobility, depression, sleep disturbances, and increased health care costs [1,2,6-9]. Currently, we know relatively little about the mechanisms that drive arthritic joint pain. This is reflected in the fact that we have remarkably few effective analgesic therapies for treating joint pain that are not accompanied by significant unwanted side effects [1,10-12]. As the life expectancy of humans continues to increase in both the developing and developed world [13,14], age-related arthritic joint pain is expected to exact an ever-increasing toll on aged individuals and society.

Arthritic joint pain most frequently occurs with movement and/or loading of the affected joint but can also be present at rest [15,16]. Movement or loading-induced joint pain is usually described as sharp and/or stabbing, whereas arthritic joint pain at rest is usually described as burning and/or throbbing, with occasional bouts of stabbing pain [15-17]. Currently, it is believed that spontaneous arthritic pain (joint pain at rest) and movement-evoked pain are largely driven by joint injury and/or inflammation, which induces both a peripheral sensitization (an increase of sensitivity of nociceptive primary afferent neurons) and central sensitization (hyperexcitability of neurons conveying nociceptive information in the central nervous system (CNS)) (see [1,18-20] for review). However, it remains largely unknown why a relatively poor correlation exists in OA between the radiologic signs of arthritis (for example, joint-space narrowing, erosive changes) and the severity of arthritic pain [1,2,21] as well as the specific mechanisms that drive spontaneous versus movement-evoked arthritic joint pain in OA. This dissociation between pain and disease progression is also observed in RA, as even therapies such as tumor necrosis factor-α inhibitors that can be quite effective at decreasing the severity of joint inflammation/swelling in RA are generally much less efficacious at attenuating RA pain [22].

One potential mechanism that may explain the dissociation between disease progression and pain in arthritis is that an active and ectopic sprouting of sensory and sympathetic nerve fibers plays a role in driving pain due to arthritis. For example, a previous study performed in young rats suggested that after injection of complete Freund adjuvant (CFA) into the temporomandibular joint of rats, significant sprouting of sensory nerve fibers occurred in the painful arthritic joint [23]. However, as aging is generally associated with a decline and deterioration of the ability of tissues to grow (with the exception of cancers) [24-27], it is not clear whether sensory or sympathetic neurons in aged animals retain the ability to undergo exuberant ectopic sprouting, and if so, whether sprouting is relevant to understanding the mechanisms that drive pain in the aged arthritic joint.

In this study, we examined the ability of sensory and sympathetic nerve fibers to undergo ectopic sprouting. The model used here was generated by unilaterally injecting CFA into the articular space of the knee joint of geriatric (27- to 29-month-old) female mice. With this model, we demonstrate that even in the arthritic knee joint of these geriatric mice, robust sprouting and formation of neuroma-like structures by both sensory and sympathetic nerve fibers was observed in all CFA-injected animals and in none of the vehicle-injected mice. These newly sprouted sensory and sympathetic nerve fibers were not only present in a higher density per unit area than was ever found in the normal aged joint, but were also found in areas of the joint that are normally poorly or not innervated.

## Materials and methods

### Animals

Experiments were performed on a total of 12 geriatric female B6/C3H mice (a generous gift from Rinat-Pfizer, San Francisco, CA, USA). Mice were received at our animal care facilities 2 months before experiments were performed. At the time of initiation of the experiments, mice were 27 to 29 months old. The mice were housed in accordance with the National Institutes of Health guidelines under specific pathogen-free conditions in autoclaved cages maintained at 22°C with a 12-hour alternating light-and-dark cycle and were given autoclaved food and water *ad libitum*. The Institutional Animal Care and Use Committee at the Minneapolis VA Medical Center approved all procedures.

### Complete Freund's adjuvant injection

A modified version of a previously validated model of arthritic inflammation of the knee joint [28] was produced by performing four intraarticular injections of complete Freund's adjuvant (CFA) dissolved in saline or saline alone (vehicle) at days 0, 7, 14, and 21 unilaterallly into the left-knee joint. At days 0 and 7, mice received 5 μl of CFA (1 μg/μl), and at days 14 and 21, mice received 10 μl of CFA (1 μg/μl). Mice treated with vehicle received the same injection volume of saline. In brief, mice were anesthetized by using 2% to 3% isoflurane mixed with air. An injection of CFA was given by using a 30-gauge, 1/2-inch needle that was fitted with cannulation tubing such that only 2.5 mm of the needle was allowed to puncture the joint. CFA or saline (vehicle) was injected through the patellar ligament into the articular space, by using the femoral condyles as a guide.

Currently, there is no well-accepted rat or mouse model of osteoarthritis (OA) simultaneously induces robust joint destruction and pain similar to that observed in humans with painful OA. One rodent model that has been used as a surrogate in terms of OA pain is CFA injection into the knee joint. As previous results from this model [29] were used to guide the human trials of anti-NGF (which proved highly efficacious in reducing human OA pain [30]), we chose to use this model to examine potential nerve sprouting in the painful inflamed knee joint. However, it is important to note that although pain behaviors were present at 4 weeks after injection, no signs were seen of significant cartilage destruction and osteophyte formation, as evaluated with histologic analysis in the knee joint. This result contrasts with the pathology reported in preclinical models of OA, including intraarticular injection of monoiodoacetate or surgical destabilization of the joint. Additionally, in our hands, CFA injections caused infiltration of CD68^+ ^monocytes/macrophages into the synovial membrane as well as tissues outside the joint, including tendons, ligaments, and surrounding muscle (data not shown). Taken together, these results suggest that the present model involves an inflammation in the synovium as well as the tendons and ligaments of the CFA-injected knee joint without signs of cartilage destruction.

### Behavioral measures of arthritic joint pain

Behavioral measures of arthritic joint pain included spontaneous pain (guarding and flinching), stimulus-evoked pain (limb use), and the ability of the animal to place weight on the arthritic limb versus the nonarthritic limb while ambulatory (dynamic weight bearing). In brief, the number of hindpaw flinches and time spent guarding were recorded as measures of spontaneous pain, as these measures mirror patients in a clinical setting with arthritis who have spontaneous pain (joint pain at rest) [15,31]. The number of spontaneous flinches and time spent guarding, representative of spontaneous nocifensive behavior, were recorded during a 2-minute observation period, as previously described [32-38]. Flinches were defined as the number of times the animal raised its hindpaw and guarding as the amount of time animals held the hindpaw aloft while not ambulatory. Based on our previous experience evaluating spontaneous pain-related behaviors in mice, three or more flinches and two or more seconds spent guarding during a 2-minute interval suggest the presence of pain-related behaviors.

Normal limb use during spontaneous ambulation in an open field was used as an indicator of stimulus-evoked pain and was scored on a scale of 5 to 0, as previously reported [34,38], where (5) is normal use, (4) is partial limp, but not pronounced, (3) is pronounced limp, (2) is limp and guarding behavior, (1) is partial nonuse of limb in locomotor activity, and (0) is complete lack of limb use. One trial of approximately 1 to 2 minutes was performed per test period. Evaluation of normal limb use during ambulation was performed to reflect the clinical condition when patients with arthritis have pain after movement of the joint [15,31].

Behavioral measures of arthritic joint pain included spontaneous pain (guarding and flinching) and stimulus-evoked pain (limb use) were performed at day 28 after CFA initial injection, whereas dynamic weight bearing was determined at day 23 after initial injection. Not enough time was available in a single day to perform all four behaviors.

Dynamic weight-bearing analysis of the arthritic limb was performed on days 0 and 23 after initial intraarticular CFA injection with the use of a Dynamic Weight Bearing device (EB Instruments, Pinellas Park, Florida, USA). Similar pedobarographic analysis is used clinically to determine the weight bearing of patients with arthritic knee joints [39]. The mouse was allowed to move freely on the electronic sensor pad within the apparatus for 5 minutes, and the sensor-captured weight-bearing information was transmitted live to a computer. By using a synchronized video recording of the test and the scaled map of the detected zones, each presumed paw detection was validated by an observer and identified as a left or right and fore- or hindpaw. Dynamic weight bearing on the affected (left) hindlimb was calculated and reported as a percentage of total weight bearing on both hindlimbs.

### Immunohistochemistry

At day 28 after initial CFA injection, mice were deeply anesthetized with carbon dioxide asphyxiation, delivered by using a compressed gas cylinder, and perfused intracardially with 20 ml of 0.1 *M *phosphate-buffered saline (PBS; pH 7.4 at 4°C) followed by 30 ml of 4% formaldehyde/12.5% picric acid solution in 0.1 *M *PBS (pH 6.9 at 4°C). Ipsilateral and contralateral knee joints were harvested after perfusion and postfixed for at least 12 hours in the same perfusion fixative. The process of postfixing, decalcification, and sectioning of the bones/joints was performed as previously described [40,41].

To qualitatively assess the changes in the density and morphology of sensory nerve fibers that innervate the knee joint, the macrophage infiltration, and neovascularization of the synovium, 20 μm-thick frozen sections of the bone/joint were processed according to our previously published procedures [35,40,41]. Frozen bone/knee-joint sections were incubated with an antibody against calcitonin gene-related peptide (CGRP, polyclonal rabbit anti-rat CGRP; 1:10,000; Sigma Chemical Co. St Louis, MO, USA; catalog no. C8198) to label primary afferent sensory nerve fibers, an antibody against neurofilament 200 kDa (NF200, chicken anti-neurofilament 200 kDa; NF200, 1:5,000; Neuromics, Edina, MN, USA; catalog no. CH22104) to label myelinated primary afferent sensory nerve fibers. Sympathetic nerve fibers were labeled with an antibody against tyrosine hydroxylase (TH, polyclonal rabbit anti-rat TH, 1:1,000; Chemicon, Billerica, MA, USA; catalog no. AB152). Sprouted nerve fibers were labeled with an antibody against growth-associated protein-43 (GAP-43, rabbit anti-GAP43, 1:1,000; Millipore, Billerica, MA, USA; catalog no. AB5220). Blood vessels were labeled with an antibody against platelet endothelial cell adhesion molecule (rat anti-mouse PECAM, 1:500, BD PharMingen, San Jose, CA, USA; catalog no. 550274). Monocytes/macrophages were indentified with an antibody against a myeloid glycoprotein (rat anti-mouse CD68; 1:2,000, AbD Serotec, Raleigh, NC, USA; catalog no. MCA1957). Additionally, four to five sequential frozen bone sections were cut at 10 μm thick and were stained with safranin-O/fast green to visualize gross pathologic changes in cartilage and bone induced by CFA.

### Quantification of nerve fiber density, sprouting, macrophage infiltration and neovascularization

Approximately 30 separate, 20-μm-thick frozen sections were obtained from the knee joint of each mouse. Three confocal images (Olympus America Inc, Center Valley, PA, USA; software v. 5.0) from different sections separated by at least 100 μm were obtained for each marker. Sections were initially scanned at low magnification (×100) to identify areas with the highest capillary or nervefiber density in the synovium. These areas in the synovium were defined as "hot spots." One image per section was acquired within the medial synovial hot spot. Whereas nerve fibers, blood vessels, and macrophages were observed throughout the inflamed synovium, neovascularization and nerve sprouting was consistently present in the synovium adjacent to the meniscus, therefore, most of the hot spots were found and quantified in this area. In the knee sections from naïve and sham mice, no evidence of "hot spots" was noted in terms of significant changes in the morphology or density of nerve fibers and blood vessels.

The volume of CFA-inflamed synovium analyzed was 315 μm (length), 315 μm (width), and 20 μm (depth). The Z-stacked images were analyzed with Image-Pro Plus v. 6.0 (Media Cybernetics, Bethesda, MD, USA), and nerve fibers and blood vessels were manually traced to determine their respective lengths. To determine whether a structure in the image was a nerve-fiber profile, simultaneous observations of the image on a high-resolution computer monitor and the section viewed in the microscope were performed. The fine focus of the microscope was used to follow the fibers across the entire thickness of the knee section. Structures were considered nerve profiles only if their signal was at least 3 times greater than background signal, if their length was > 20 μm, and if they displayed the presence of dilatations (varicosities). Small dot-like structures were not counted, even though these structures likely represented cut fibers. Nerve sprouting or neovascularization was reported as density of nerve fibers or blood vessels per volume of synovium (mm/mm^3^) [40]. CD68^+ ^macrophages were quantified in each layer of the inflamed synovium from z-series images (×400 magnification) of each field of view by using Imaris Pro Software v. 6.0 (Bitplane AG, South Windsor, CT, USA). Only CD68^+ ^cells that displayed visible nuclei, as determined by counterstaining with DAPI, were counted. Only CD68^+ ^macrophages/monocytes located in the normal or inflamed synovium were quantified. Macrophages/monocytes were defined as CD68^+ ^profiles that were distant from any bone surface, whereas osteoclasts were defined as CD68^+ ^profiles that were directly apposed to mineralized bone [42,43].

Data from at least three slices per knee joint were recorded and averaged. The total number of CD68^+ ^macrophages in the synovium was reported. Total volume of the synovium (naïve and inflamed) was calculated by tracing the area of the synovium and multiplying this area by the thickness of the section (20 μm).

Density of nerve fibers, PECAM^+ ^blood vessels and CD68^+ ^monocytes/macrophages in the femoral periosteum were calculated within the distal metaphysic, as significant neural remodeling was observed in this region. The area evaluated in frozen sections was within a 1.0-mm-long region, which started 0.5 mm below the distal femoral growth plate. For frozen sections, the average volume of periosteum analyzed was 315 μm (length), 50 μm (width), and 20 μm (depth). The confocal images were viewed on a high-resolution monitor, and the length of nerve fibers was determined by manually tracing the nerve or blood vessel profile by using Image Pro Plus v. 6.0 image analysis software (Media Cybernetics, Bethesda, MD, USA). Nerve sprouting or neovascularization was reported as density of nerve fibers or blood vessels per volume of periosteum (mm/mm^3^). Data from at least three slices per knee joint were recorded and averaged. CD68^+ ^macrophages were quantified in both layers of the periosteum from z-series images of each field by using Imaris Pro-Software v. 6.0 (Bitplane AG, South Windsor, CT, USA). The density of CD68^+ ^macrophages in the periosteum was reported as number of CD68^+ ^macrophages per volume of periosteum.

To determine whether changes were present in the density of nerve fibers or the formation of neuroma-like structures, frozen sections were examined at low magnification with an epifluorescent microscope. Areas that were examined included the lower half of the femur and the upper half of the tibia and included the mineralized bone, bone marrow, cartilaginous knee joint, synovium, periosteum, and knee-joint ligaments. In general, areas that contained sprouting of nerve fibers and neuroma-like structures were remarkably different from nerve fibers in the normal bone and joint in terms of morphology and density. For example, a neuroma-like structure was defined as a disordered mass of axons (CGRP^+^, NF200^+^) that has an interlacing and/or whirling morphology, a size that is at least 10 μm thick by 70 μm long (> 10 individual axons) [44-46], and that we have never observed in any bone or joint compartment, including the synovium or periosteum of normal joint or bone [41,47-49].

### Statistics

A *t *test was used to compare the behavioral measures of spontaneous pain (guarding and flinching) and dynamic weight bearing. The Mann-Whitney Rank Sum Test was used to compare the stimulus-evoked pain (limb use). One-way ANOVA followed by the Dunnett *post hoc *test was used for the immunohistochemical measures between the experimental groups. Significance level was set at *P *< 0.05. In all cases, the investigator responsible for behavioral testing, plotting, measuring, and counting was blind to the experimental situation of each animal.

## Results

### CFA injection into the knee joint of geriatric mice produces significant pain-related behaviors

**Figure 1:**
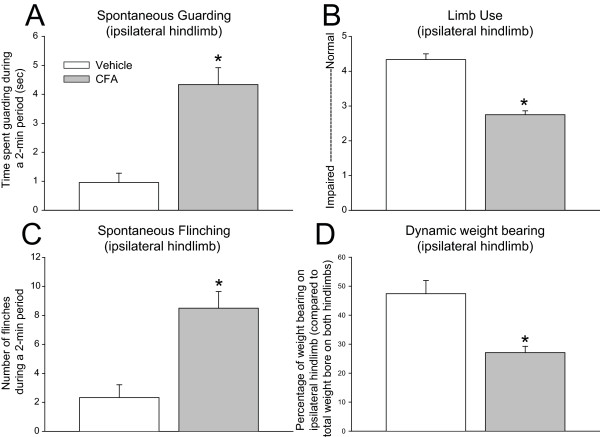
**A complete Freund's adjuvant model of inflammatory arthritis induces significant and long-term pain behaviors**. The spontaneous time spent guarding **(A)**, and the number of spontaneous flinches **(C) **in CFA-treated mice were significantly greater than those in vehicle-treated mice at 28 days after initial CFA injection. In addition, limb use **(B) **and dynamic weight bearing of the ipsilateral hindlimb **(D) **declined significantly after the initial CFA injection and continued to decline through the 24-day experimental period. Bars represent the mean of at least five mice ± SEM. *P < 0.05 versus mice that received intraarticular injections of saline.

Pain-related behaviors were evaluated in CFA-injected animals as compared with saline-treated animals. Assessment of arthritic joint pain-related behaviors, including spontaneous guarding and flinching, and limb-use analysis of the hindlimb was performed on day 28 after initial intraarticular CFA injection, and dynamic weight-bearing analysis was performed on day 23 after initial intraarticular CFA injection (Figure 1). At day 28 post-injection, CFA-treated mice exhibited a greater time spent guarding, and number of flinches as compared to those observed in vehicle-treated mice (Figure 1). Additionally, the evaluation of ambulatory pain revealed that the mean score of limb use was reduced from a 4.3 ± 0.1 in vehicle-treated mice to 2.7 ± 0.11 in CFA-treated mice at day 28 after cell injection. Similarly, CFA-induced arthritis resulted in a significant reduction in dynamic weight bearing of the CFA-injected hindlimb, as compared with the vehicle-treated hindlimb (Figure 1).

### CFA injection into the knee joint of geriatric mice produces joint edema, infiltration of macrophages, and aberrant neovascularization in the synovium

Repeated injections of CFA resulted in significant edema within the knee joint at day 28 after initial injection. In all CFA-injected mice, the inflammation was restricted to the ipsilateral joint with no evidence of inflammation present in the contralateral hindlimb.

**Figure 2:**
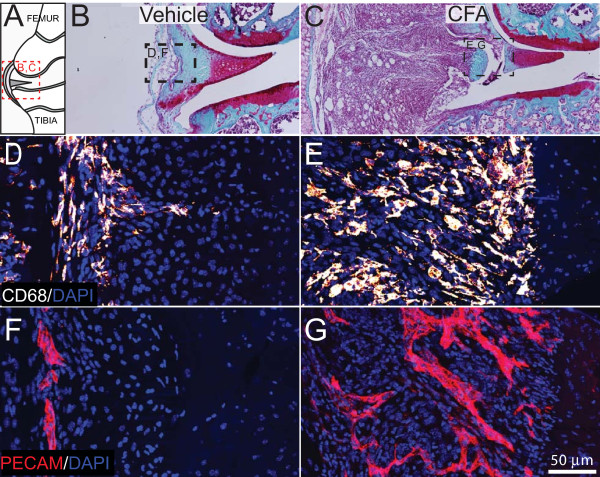
**CFA injection into the geriatric knee joint induces synovial inflammation, macrophage infiltration, and neovascularization**. **(A) **Schematic of the frontal view of a cross-sectioned mouse knee joint, indicating the location of the major cellular and structural changes occurring in the arthritic knee joint. **(B-G) **Longitudinal cross sections (10 μm thick) of the knee joint stained with safranin-O/fast green display the histopathologic changes in the synovium and capsule 28 days after initial injection of complete Freund's adjuvant (CFA). In the vehicle-injected mice, the synovium is very thin and relatively intact **(B)**, whereas in CFA-injected mice, an extensive inflammation in the synovial membrane and thickening of the joint capsule are found **(C)**. The outlined boxes in B and C illustrate the region from which the subsequent confocal images were obtained. Representative confocal images of CD68^+ ^macrophages (**D, E**, red/orange), DAPI-labeled nuclei (blue), and a vascular endothelial marker, PECAM^+ ^(F, G, red) in vehicle-injected **(D, F) **and CFA-injected **(E, G) **mouse knee-joint sections (20 μm-thick). Injections of CFA induce a significant infiltration of CD68^+ ^macrophages into the synovium **(E)**, as compared with vehicle-treated mice **(D)**. In vehicle-injected mice, a low-level vascularization by PECAM^+ ^vessels is observed in the synovial space of the knee joint **(F)**. In contrast, in CFA-treated knee joints, a significant number of PECAM^+ ^vessels have developed and have an enlarged and disorganized morphology **(G)**, as compared with vehicle-treated mice.

CFA-induced changes in structures of the knee joint, including bone, synovium, and periosteum were examined with histology (safranin-O/fast green staining) and immunonohistochemistry. At day 28 after initial injection, the repeated intraarticular injection of vehicle did not induce a visible inflammatory response in the synovium (Figure 2B) or major changes in the structure of articular cartilage (femur and tibia). The knee sections from vehicle-treated mice and mice without injection (naïve) were similar in appearance. In contrast, repeated injections of CFA into the knee induced an extensive inflammation in the synovial membrane and thickening of the joint capsule (Figure 2A, C). Repeated CFA injections caused the infiltration of many CD68^+ ^monocytes/macrophages into the synovial membrane (Figure 2E) and tissues outside the joint (periarticular inflammation) including tendons, ligaments, and surrounding muscle (data not shown). Knee sections stained with antibody that recognizes endothelial cells found in blood vessels (PECAM), showed few small blood vessels in the synovial membrane (Figure 2F) of vehicle-treated mice. In contrast, CFA injection induced an increase in the number of PECAM^+ ^blood vessels, which were different from those that vascularize normal synovium, as they were thicker and exhibited disorganized, nonlinear morphology (Figure 2G).

**Figure 3:**
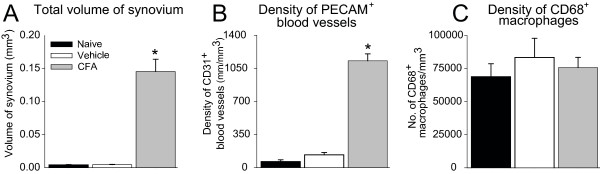
**Histograms showing the changes induced by CFA injection into the geriatric knee joint**. Volume of synovium (mm^3^) **(A)**, density of PECAM^+ ^blood vessels **(B)**, but not the density of CD68^+ ^macrophages per unit area **(C) **were increased 4 weeks after initial CFA injection. Each bar represents the mean ± SEM. *P < 0.05 versus mice that received intraarticular injections of saline as vehicle.

Quantitative image analysis revealed an increase in the volume of the synovial membrane during arthritis from 0.0044 ± 0.0003 mm^3 ^in vehicle-treated knees to 0.1454 ± 0.0186 mm^3 ^on Day 28 in CFA-treated knees (Figure 3A). A significant increase in the density of PECAM^+ ^blood vessels was present in CFA-treated knees as compared with vehicle-treated or naïve knees (Figure 3B). The CD68^+ ^monocyte/macrophage density in the synovium of CFA-treated knees was not significantly different as compared with the synovium from vehicle-treated knees (Figure 3C). When the total number of CD68^+ ^monocytes/macrophages in the synovium was calculated as the product of cell density and synovial volume, the normalized total cell number in the synovium was significantly greater in CFA-treated mice (11 007 ± 1,125 CD68^+ ^profiles) as compared with vehicle-treated (365 ± 63 CD68^+ ^profiles) or naïve mice (301 ± 42 CD68^+ ^profiles).

### CFA injection into the knee joint of geriatric mice produces sprouting and formation of neuroma-like structures by sensory and sympathetic nerve fibers in the synovium and periosteum

**Figure 4:**
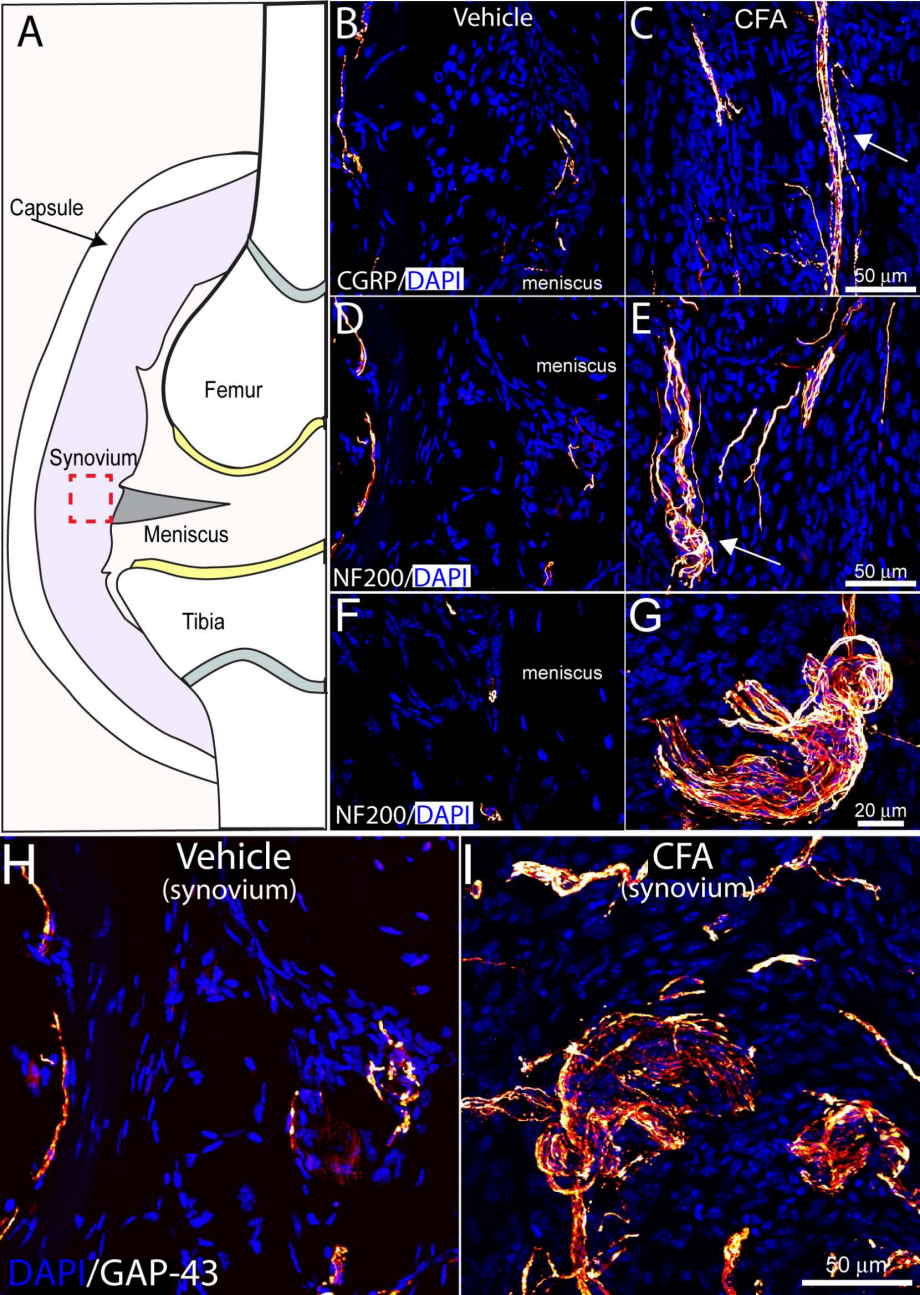
**Sensory nerve fiber sprouting and formation of neuroma-like structures in the painful geriatric arthritic knee joint**. Schematic of a frontal view of a cross-sectioned mouse knee joint **(A)**. The red square illustrates the synovial region from which the confocal images were obtained. Representative confocal images of calcitonin gene-related peptide (CGRP^+^), neurofilament 200-kDa (NF200^+^) sensory nerve fibers (yellow/orange), and growth-associated protein (GAP43; marker of fibers undergo regeneration, yellow/orange) and DAPI-labeled nuclei (blue) in knee-joint sections (20 μm thick) of vehicle-injected **(B, D, F, H) **and CFA-injected **(C, E, G, I) **mice. In vehicle-injected mice, a low-level, regular pattern of innervation by CGRP^+ ^and NF200^+ ^fibers is observed in the synovial space of the knee joint. Twenty-eight days after the initial CFA injection, a significant number of CGRP^+^, NF200^+^, and GAP43^+ ^nerve fibers have sprouted and have a disorganized appearance, as compared with vehicle-injected mice. Note that CGRP^+^, NF200^+^, and GAP43^+ ^sprouted nerve fibers are localized in the synovium and are not observed in the meniscus of the joint. Furthermore, formation of neuroma-like structures occurred in the synovium of the geriatric mice injected with CFA **(G)**.

**Figure 5:**
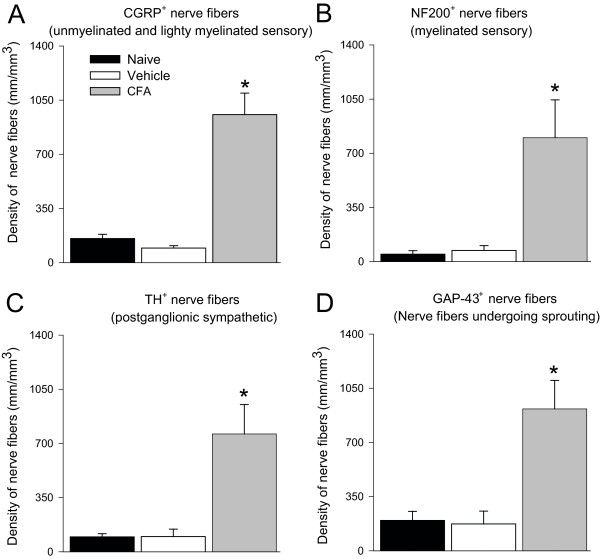
**Histograms showing an increase in the density of sensory and sympathetic nerve fibers in the geriatric arthritic knee joint**. The length of calcitonin gene-related peptide (CGRP^+^, **A**) sensory nerve fibers, neurofilament 200 kDa (NF200^+^, **B**) sensory nerve fibers, tyrosine hydroxylase (TH^+^, **C**) postganglionic sympathetic nerve fibers and growth-associated protein-43^+ ^(GAP-43^+^, **D**) nerve fibers undergoing sprouting in the synovium were obtained by manually tracing the nerve fibers in a determined volume of synovium. Bars represent the mean of at least three mice ± SEM. *P < 0.05 versus mice that received intraarticular injections of saline.

CGRP^+ ^(sensory, Figure 4B), NF200^+ ^(sensory, Figure 4D), TH^+ ^(sympathetic), and GAP43^+ ^(nerve fibers undergoing sprouting; Figure 4I) nerve fibers were evident only at low levels in the synovial-meniscal interface from vehicle-treated knee sections (Figure 4B, D, F, H). In contrast, 28 days after the initial CFA injection, a robust sprouting of CGRP^+^, NF200^+^, TH^+^, and GAP43^+ ^nerve fibers was observed within the synovium of the inflamed joint (Figure 4C, E). These newly sprouted nerve fibers are found in higher density (Figure 5) and have a highly disorganized appearance as compared with the primarily linear morphology of these nerve fibers in the synovium of vehicle-treated or naïve mice. Whereas 100% of CFA-injected mice showed significant sprouting of CGRP^+^, NF200^+^, GAP43^+^, and TH^+ ^nerve fibers, five of six mice had two to three neuroma-like structures in the synovium (Figure 4G). Only one of six mice displayed a large neuroma (approximate thickness of 50 μm). These neuroma-like structures appear as a disordered mass of blind-ending axons that have an interlacing or whirling morphology and are never observed in vehicle-treated mice (Figure 4F).

**Figure 6:**
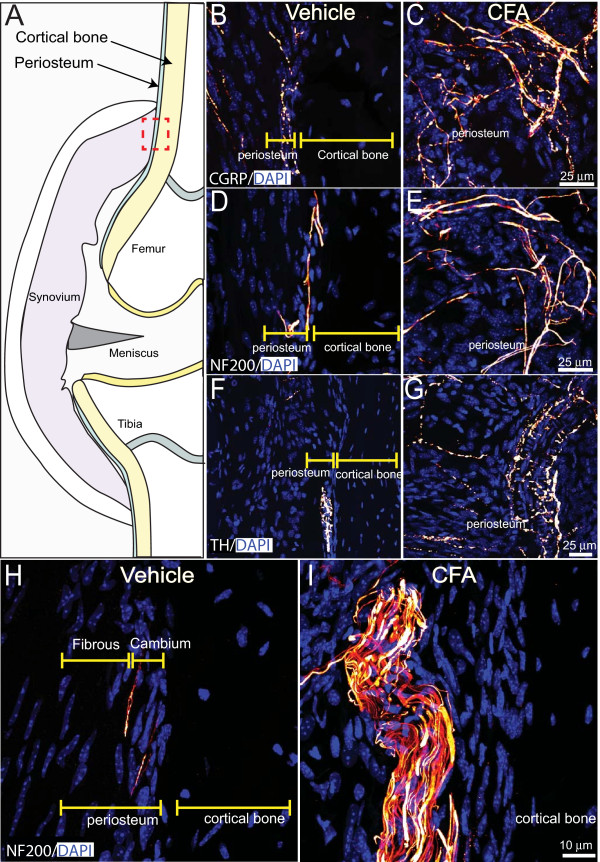
**Intraarticular injection of CFA induces nerve sprouting and formation of neuroma-like structures in the periosteum**. **(A) **Schematic of the frontal view of a cross-sectioned mouse knee joint illustrating the periosteal region from which the confocal images were obtained (red square). Representative confocal images of CGRP^+ ^and NF200^+ ^sensory nerve fibers (yellow/orange), TH sympathetic nerve fibers (yellow/orange), and DAPI labeled nuclei (blue) in 20 μm-thick knee sections from vehicle-injected **(B, D, F, H) **and CFA-injected **(C, E, G, I) **mice. In vehicle-injected mice, a low-level, regular pattern of innervation by CGRP^+^, NF200^+^, and TH^+ ^nerve fibers is observed in the synovial space of the knee joint. Twenty-eight days after the initial CFA injection, a significant number of CGRP^+^, NF200^+^, and TH^+ ^nerve fibers have sprouted and have a disorganized appearance as compared with vehicle-injected mice. Furthermore, a formation of neuroma-like structures by NF200^+ ^nerve fibers was seen in the femoral periosteum of the geriatric mice injected with CFA **(I)**.

To determine whether the nerve sprouting induced by the arthritis was present in other knee-joint compartments, we qualitatively examined cartilage, synovium, cortical bone, trabecular bone, marrow space, ligaments, and periosteum. Sensory and sympathetic nerve fibers were present in these compartments, as previously described [48-54], however, a noticeable change in the density of sensory and sympathetic nerve fibers and/or neuroma formation was observed only in the synovium and periosteum of inflamed knee joints. Confocal micrographs of metaphyseal periosteum preparations from vehicle-treated mice revealed that CGRP^+ ^(Figure 6B) and NF200^+ ^(Figure 6D) nerve fibers normally have a linear and bifurcating pattern. In contrast, periosteal preparations from CFA-treated mice displayed an aberrant and disorganized morphology of CGRP^+ ^(Figure 6C), NF200^+ ^(Figure 6E) sensory nerve fibers. The majority of TH^+ ^sympathetic fibers in the periosteum of vehicle-treated mice possessed a unique "corkscrew" morphology that appeared to wrap around small blood vessels in the periosteum (Figure 6F). In contrast, in the periosteum of CFA-treated mice TH^+ ^nerve fibers displayed a highly pathologic and disorganized pattern of innervation as well as appearing to wrap large blood vessels in a tight corkscrew-like fashion (Figure 6G). Additionally, five of six mice treated with CFA had two to three neuroma-like structures in the periosteum (Figure 6I).

Quantitative analysis of the density of nerve fibers (expressed as length of nerve fibers/mm^3 ^of femoral periosteum) revealed that CFA-induced knee arthritis resulted in a significant increase in the density of CGRP*^+ ^*fibers as compared with mice treated with vehicle (3,314.9 ± 377 versus 2,085 ± 380 mm/mm^3^; *P *< 0.05). Likewise, the density of NF200*^+ ^*sensory nerve fibers in the femoral periosteum was significantly increased in CFA-treated mice as compared with vehicle-treated (3,944 ± 1,176 versus 1,144 ± 160 mm/mm^3^; *P *< 0.05). Similarly, the density of postganglionic TH^+ ^sympathetic nerve fibers increased significantly in the femoral periosteum of mice treated with CFA as compared with vehicle-treated mice (3,392 ± 291 versus 1,020 ± 64 mm/mm^3^; *P *< 0.05). Finally, the density of nerve fibers undergoing sprouting (GAP43^+^) also increased significantly in the femoral periosteum of mice that received intraarticular injections of CFA as compared with mice injected with vehicle (4,677 ± 805 versus 1,596 ± 261 mm/mm^3^; *P *< 0.05). The density of nerve fibers in the periosteum of vehicle-treated mice was not significantly different when compared with femoral periosteum of naïve mice (data not shown).

## Discussion

### Age, arthritis, and joint pain

Although chronic joint pain can be caused by a diverse group of injuries and disorders, what most forms of arthritis have in common is that the prevalence increases with age and is frequently accompanied by significant pain and impairment of physical function [1-6,55]. Unfortunately, management of age-related skeletal pain can be difficult, as current therapies are often not fully effective and have a high incidence of dose-limiting side effects [10-12]. Thus, the major therapies used to treat arthritic joint pain are nonsteroidal antiinflammatory drugs (NSAIDs) and opioids, which have remained virtually unchanged for decades [11,12].

### Disease progression and joint pain

Currently, our understanding of what drives arthritic joint pain is that joint injury, inflammation, and/or deterioration of the joint causes an increased responsiveness of the joint nociceptors (peripheral sensitization) and of the nociceptive neurons of the CNS (central sensitization) (see [1,18,19] for review). It has been reported that as joint and adjacent bone are injured because of inflammation, nerves that innervate the bone are first activated and sensitized by factors such as bradykinin, prostaglandin E_2_, prostaglandin I_2_, serotonin, substance P, galanin, neuropeptide Y, and nociceptin, which are released by stromal/inflammatory/immune cells in the injured joint (see [18,19] for review). As the joint continues to deteriorate, these "sensitized" nerve fibers then become activated when noxious or nonnoxious mechanical stimuli are applied to the joint (see [1,18-20] for review). As the cartilage deteriorates to the point at which it is no longer intact, bone-on-bone interactions can occur, which may induce direct mechanical stimulation of these sensitized nerve fibers that are present in the adjacent bone [1,20]. Paradoxically, if this were the only mechanism driving arthritic joint pain, a clear correlation should appear between joint deterioration/destruction and joint pain. However, in both trauma and age-related osteoarthritis, a poor correlation is noted between the extent of joint destruction and the frequency and severity of joint pain [3,21,56].

Although these mechanisms certainly contribute to arthritic joint pain, the present data demonstrate that the nerve fibers that innervate the joint are not simply static structures that respond to changes in the joint and bone but rather can undergo a remarkable reorganization in terms of altered morphology, increase in the density of nerve fibers per unit area, and sprouting into areas of the joint that are normally poorly innervated. The populations of nerve fibers that are undergoing sprouting include CGRP^+ ^and NF200^+ ^sensory nerve fibers that correspond to unmyelinated/thinly myelinated and thinly myelinated sensory nerve fibers, respectively, as well as TH^+ ^postganglionic sympathetic nerve fibers. Previous human [57-69] and rodent [23,35,46,70-74] studies have shown that inappropriate remodeling of sensory and sympathetic nerve fibers can be associated with skeletal [23,35,46,62,63,68,69,71] and nonskeletal pain [57,60,64-67,72-74]. As we observed a similar remodeling of sensory and sympathetic nerve fibers in geriatric arthritic knee joints, this pathologic reorganization of nerve fibers would then set in place a neuroanatomic substrate that could drive movement and resting arthritic joint pain.

In addition to the robust sprouting of sensory and sympathetic nerve fibers in the knee joints of the geriatric mice that received injections of CFA, we observed the appearance of neuroma-like structures in the synovium and femoral periosteum. Previous preclinical and clinical studies have shown that injury to peripheral nerves due to trauma, amputation, compression, or surgery can lead to the formation of painful neuromas [58,59,75,76], which have a morphology similar to the neuroma-like structures observed in the present study. In humans, these nonmalignant neuromas frequently cause chronic and severe pain [58,59,77], produce spontaneous ectopic discharges [76,78,79], in part by upregulation of sodium channels [59,80,81], and are largely refractory to medical treatment [59]. Whether these neuroma-like structures in the geriatric arthritic knee joints also show an upregulation of sodium channels and produce spontaneous discharges is unknown, but these structures could partially explain the phenomenon of spontaneous arthritic joint pain (pain at rest), as movement would not be required for these spontaneous ectopic and painful discharges to occur.

In addition to the reorganization of nerve fibers in the knee joints of the geriatric mice that received injections of CFA, we found a significant increase in the total number of CD68^+ ^macrophages/monocytes in the synovium (as the inflamed synovium is much larger than the naïve or sham-operated synovium) but not changes in the density per unit areas of these inflammatory cells. Although the lack of change in macrophage/monocyte density in relation to the difference in total numbers of these cells between the groups is unknown, it is possible that that age-related loss in the macrophage infiltration and function may explain this finding [82].

### Sprouting of sensory and sympathetic nerve fibers in the geriatric knee joint

In general, neuroplasticity is thought to decline with age. For example, a previous study showed that the rate of axonal regeneration as determined by axonal transport of radiolabeled proteins after sciatic nerve injury is slower in 28-month-old rats as compared with 2- and 10-month old rats [27]. In 24-month-old mice, the number of regenerating myelinated axons in tibial fascicles is significantly reduced (~50% decrease) compared with 6-month-old mice after crushing at the sciatic nerve [25]. Additionally, it has been shown that the collateral sprouting of sensory and sympathetic axons after injury is 50% less in aged rats as compared with young rats [24,26].

In contrast to these studies, our results indicate that sensory and sympathetic nerve fibers that innervate the knee joints of geriatric mice not only retained the capacity to undergo a robust and inappropriate sprouting but they also formed neuroma-like structures after inflammation or injury. One potential explanation as to why the neuronal sprouting of sensory and sympathetic nerve fibers is so robust in the aged knee joint is that an age-related maintenance occurs in the synthesis and release of growth factors from skeletal tissues. In support of this hypothesis, the expression of different growth factors, including bone morphogenetic protein-2, bone morphogenetic protein-7, insulin-like growth factor-1, leptin, tumor necrosis factor-α, and transforming growth factor-β are maintained and/or increased in the intervertebral discs in aged rabbits and humans [83-85]. Additionally, maintenance in the expression and responsiveness of receptors expressed by the nerves innervating the joints may promote the nerve sprouting and neuroma formation observed in the present study.

A key unknown is the factor(s) driving the sprouting and neuroma formation of sensory nerve fibers and ultimately the pain in the painful inflamed joint. Previous reports exhaustively demonstrated that in both the developing and adult animal, nerve growth factor can induce marked sprouting of sensory and sympathetic nerve fibers (see [86] for review). Furthermore, previous studies in our laboratory, using a mouse monoclonal antibody against NGF (anti-NGF), showed that sustained administration of anti-NGF results in a marked reduction of sprouting and neuroma-like formation by CGRP^+^, TH^+^, and NF200^+ ^nerve fibers in the tumor-bearing bone, irrespective of whether the tumor synthesizes NGF [35,46,71]. These data, together with significant literature suggesting that macrophages, neutrophils, endothelial cells, T-lymphocytes, and fibroblasts can all express significant levels of NGF (see [87] for references), suggest that NGF may be a key factor driving the reorganization of sensory and sympathetic nerve fibers in inflamed knee joints from geriatric mice.

## Conclusions

In this study, CFA-induced inflammation of the geriatric knee joint resulted in significant pain, robust nerve sprouting, and formation of neuroma-like structures in the synovium and periosteum by both sensory (CGRP^+ ^and NF200^+^) and sympathetic (TH^+^) nerve fibers. Interestingly, these sprouting nerve fibers were present at a higher density and had unique morphology that is never observed in the normal nonpainful geriatric knee joint. Although robust sprouting and neuroma formation are clearly present at 28 days after CFA injection, it remains to be determined when this nerve sprouting and neuroma formation first begins.

As sprouting and reorganization of sensory and sympathetic nerve fibers has been observed in other chronic pain states in both animals and humans [23,35,46,57-71] and has been correlated with the establishment of a chronic pain state, the present data suggest that ectopic sprouting may be involved in the generation and/or maintenance of arthritic pain in the aged joint. Understanding the specific factors that promote the observed nerve sprouting, defining the contribution that ectopic nerve sprouting plays in driving arthritic joint pain, and determining whether this ectopic nerve sprouting is transitory or permanent in nature may provide mechanistic insight and novel pharmacologic targets for more effective control of age-related joint pain.

## Abbreviations

CFA: complete Freund adjuvant; CGRP: calcitonin gene-related peptide; CNS: central nervous system; DAPI: 4',6-diamidino-2-phenyl-indole; GAP43: growth-associated protein-43; NSAIDs: nonsteroidal antiinflammatory drugs; NF200: neurofilament 200 kDa; PBS: phosphate-buffered saline; SEM: standard error of the mean; TH: tyrosine hydroxylase; TNF-α: tumor necrosis factor-alpha.

## Competing interests

None of the authors of this study claims a conflict of interest.

## Authors' contributions

JMJA and PWM participated in the conception and design of the experiments, in the analysis and interpretation of the data, and in the writing of the manuscript. Both authors approved the final version of the manuscript to be published.
